# Health Information Literacy as a Determinant of Digital Self-Efficacy

**DOI:** 10.3928/24748307-20250915-01

**Published:** 2026-01

**Authors:** Tuuli T. Turja

**Affiliations:** a Tampere University, Faculty of Social Sciences, Tampere, Finland.

## Abstract

This brief report examines the association between health information literacy and digital self-efficacy (DSE) in patients with multiple sclerosis or epilepsy (pwMS/E). The focus is specifically on critical and risk-conscious aspects of health information literacy. The study used survey data (*N* = 287) collected from pwMS/E. The analysis comprises a principal component analysis and a regression analysis. In a model controlling age, gender and subjective anxiousness and/or depression, a high level of health information literacy was associated with higher DSE. The model explained over one-third of the variance in DSE. The found association between health information literature and DSE in a Finnish sample of pwMS/E increases the generalizability of the findings of the previous review study. Chronically ill patients with adequate health information literacy are confident in their ability to manage their illness, including on digital platforms.

Health information literacy (HIL) refers to the ability to find, interpret, and produce health-related information. It involves not only individual skills but also the availability of information (Massey et al., 2017; Wagner et al., 2022). Health information-literate individuals know where to find relevant information about their conditions and have access to that information. One important dimension of HIL is critical literacy, that is, the ability to evaluate information critically and obtain information from reliable sources (Ishikawa et al., 2008; Yoon et al., 2022). Risk literacy, which is another important dimension in the health care context, refers here to how people understand risks involving their well-being, conditions and treatment (Chakrabarty & Mishra, 2024; Donovan et al., 2014).

In their systematic review of functional, communicational and critical health literacy, Xu et al. (2018) found that communicational and critical health literacies are important in improving self-efficacy among patients with diabetes. The current brief report contributes to the existing literature by focusing, first, on critical and risk-conscious HIL among a different group of chronic patients and, second, on digital self-efficacy (DSE) in personal health management. The latter includes a research task of extracting DSE measure from an existing eHealth literacy scale in order to isolate a conceptually coherent construct from a broader instrument.

DSE, as a meta-cognitive belief specific to the digital domain, refers to individuals' perceptions of their competence and confidence in using and learning digital services, applications, and devices. (Turja, 2021; Xu et al., 2018). Due to the digitalization of health care services, DSE is also a theme in studies of self-efficacy among patients and eHealth use. Patients with multiple sclerosis or epilepsy (pwMS/E) are used as an exemplary group of people who have been diagnosed with a chronic illness. They make a particularly relevant sample because both conditions require long-term self-management and education (Huang et al., 2025; Spreadbury et al., 2022). Based on Xu et al. (2018), the study sets a hypothesis that pwMS/E who report more advanced HIL also score higher in DSE.

## Methods

The study used survey data (*N* = 287) collected from pwMS/E in Finland in 2023 using member databases. The sample of MS patients (*n* = 186) was collected in collaboration with the Finnish MS Society and the sample of patients with epilepsy (*n* = 101) in collaboration with the Finnish Pensioners' Federation and the Finnish Epilepsy Association. Besides the primary data collection method of an electronic questionnaire, a paper-form questionnaire was completed by 18 patients at an outpatient clinic to complement the sample. The statistics did not differ between the paper and electronic questionnaires.

The questionnaire collected sociodemographic and health-related background information from the respondents as well as using the validated Multidimensional Readiness and Enablement Index for Health Technology (READHY), including the eHealth literacy scale (eHLQ) (Kayser et al., 2019). The responses to the READHY items were given on a Likert scale ranging from *strongly disagree* (1) to *strongly agree* (4).

### Extracting the Dependent Variable from the Data

The eHLQ scale of 35 items was the main focus of this study design. The items were extracted by principal component analysis. First, attempts were made to fit the data into the eHLQ's original seven dimensions of individual competence, interaction between the patient and the digital services, and individual experience with digital systems or services (Kayser et al., 2018). However, since the loadings for components beyond the first three were negligible in the relatively small amount of data, the final analysis was limited to three components, ensuring a more robust and interpretable solution. Fifteen iterations were needed to achieve convergence.

The first component included 14 statements of individual competence, such as “I easily learn to use new…,” “I quickly learn how to find my way…,” and “Overall, I understand how… (Kayser et al., 2018). In other words, the respondents responded according to how competent they believed they were in using health technology. The second and third components included statements that were more dependent on the service provider, namely, the experience of digital services, the safety of such services, and the interaction between the patient and the digital services.

The analysis of this study focused on the first component, and the perceptions of own digital competence using digital health services were interpreted as DSE in personal health management. The mean variable for the 15 items showed excellent internal consistency (*α* = 0.94).

### Independent Variable

HIL was measured by two statements with different focal points. The first measured health risk literacy of the chronically ill: “I am able to evaluate information about the risks associated with various treatments relevant to my personal health,” (Chakrabarty & Mishra, 2024), whereas the second item emphasized the dimension of critical information literacy: “I consider the credibility of the information concerning my illness or health.” (Ishikawa et al., 2008; Yoon et al., 2022). The items were rated on a 5-point scale ranging from 1 (*never*) to 5 (*often*). The mean variable for the two items showed internal consistency (*α* = 0.47) sufficient for the analysis.

### Statistical Analysis

The statistics are presented in percentages, means (*M*), standard deviations (*SD*), and coefficients of internal consistency (Cronbach's α). A principal component analysis for the dependent variable DSE used oblimin rotation with Kaiser normalization. In the multivariate analysis, DSE in personal health management was examined for its associations with HIL, controlling for age, gender and subjective anxiousness and/or depression (**Table 1**). In the linear regression analysis, standardized betas (β) and standard errors are reported, along with adjusted R-squared that indicates the explanatory power of the model.

**Table 1:**
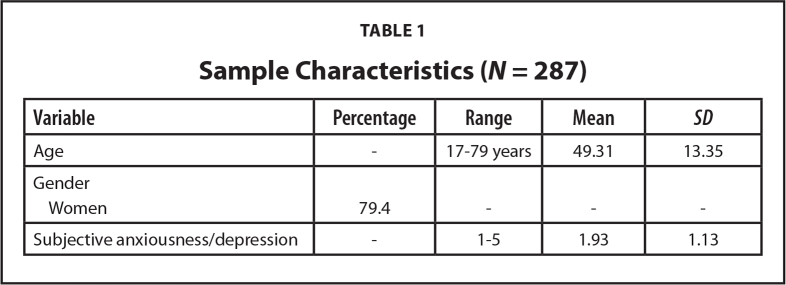
Sample Characteristics (*N* = 287)

**Variable**	**Percentage**	**Range**	**Mean**	***SD***

Age	-	17–79 years	49.31	13.35

Gender				
Women	79.4	-	-	-

Subjective anxiousness/depression	-	1–5	1.93	1.13

### Ethical Statement

The study complies with the regulations of the World Medical Association Declaration of Helsinki and the European General Data Protection Regulation, and it was conducted according to good research practice following the European Code of Conduct for Research Integrity. Informed consent from the participants was taken at the beginning of the survey in accordance with the relevant guidelines and regulations, approved by The Ethics Committee of the Tampere Region, Finland (id 18/2021). The participants' informed consent included the publication of anonymized responses.

## Results

First, a principal component analysis was conducted to identify the component of DSE in personal health management (**Table A**). The first component in the output had extraction sums of squared loadings accounting for 41.9% of the variance, indicating that this component, DSE, was more distinct than the secondary components. The mean variable calculated for DSE showed relatively high self-efficacy in the Finnish pwMS/E data (range = 1–4; *M* = 3.06; *SD* = 0.57).

**Table A:**
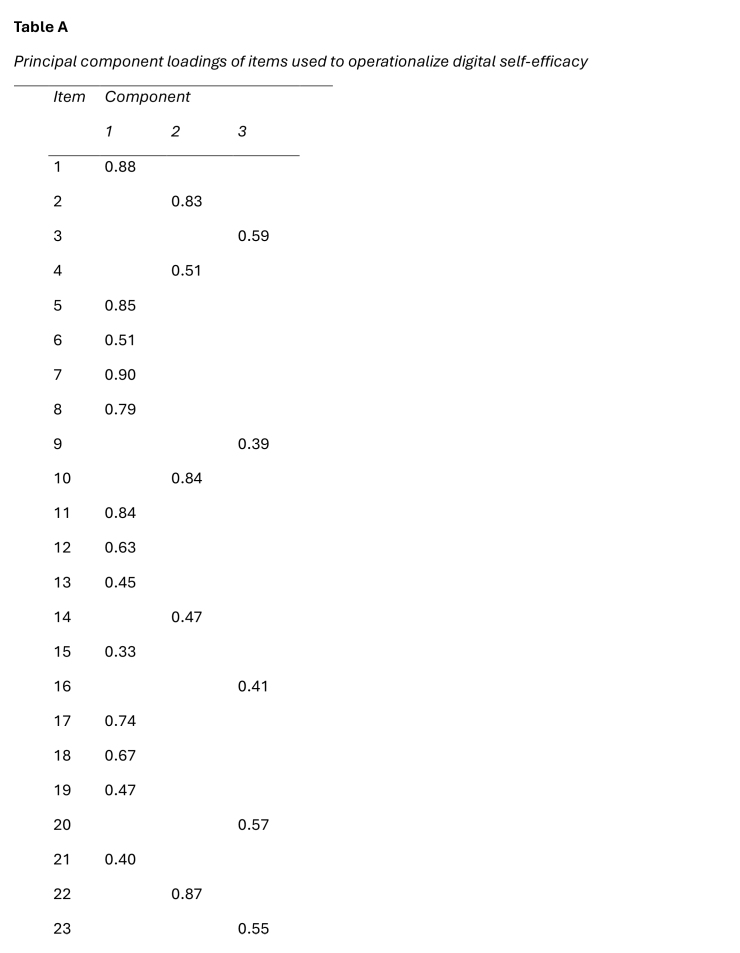
Principal component loadings of items used to operationalize digital self-efficacy

*Item*	*Component*		
	*1*	*2*	*3*
1	0.88		
2		0.83	
3			0.59
4		0.51	
5	0.85		
6	0.51		
7	0.90		
8	0.79		
9			0.39
10		0.84	
11	0.84		
12	0.63		
13	0.45		
14		0.47	
15	0.33		
16			0.41
17	0.74		
18	0.67		
19	0.47		
20			0.57
21	0.40		
22		0.87	
23			0.55

**Table:**
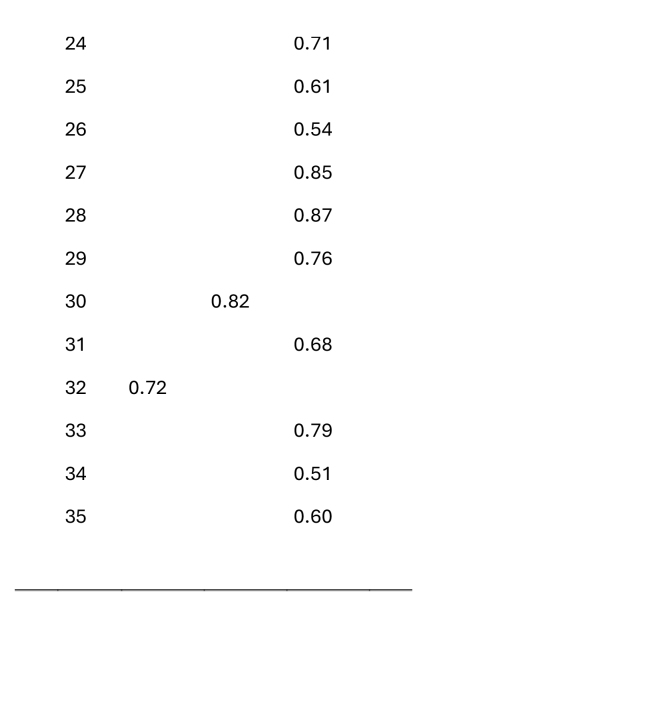


24			0.71
25			0.61
26			0.54
27			0.85
28			0.87
29			0.76
30		0.82	
31			0.68
32	0.72		
33			0.79
34			0.51
35			0.60

Second, DSE in personal health management was examined in relation to the primary explanatory variable of HIL, along with covariates including age, gender, and subjective anxiousness and/or depression. DSE stood out as a strong positive correlate to HIL, and the association was consistent among patients of different ages and genders. As an additional finding, the respondents who reported less anxiousness or depression had a higher probability of scoring higher in DSE. Together, HIL and subjective anxiousness or depression explained a sizable 36% of the variation in the patients' DSE. The results are shown in **Table 2**.

**Table 2:**
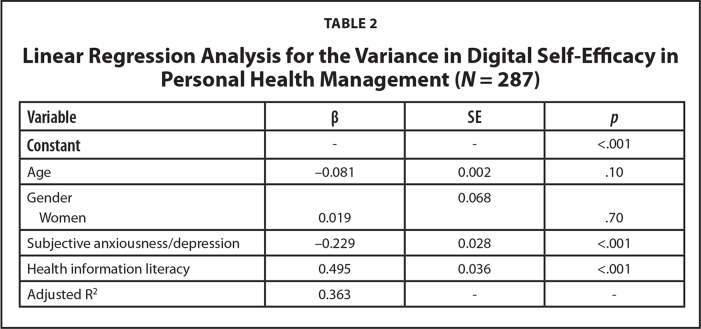
Linear Regression Analysis for the Variance in Digital Self-Efficacy in Personal Health Management (*N* = 287)

**Variable**	**β**	**SE**	***p***

**Constant**	-	-	<.001

Age	−0.081	0.002	.10

Gender		0.068	
Women	0.019		.70

Subjective anxiousness/depression	−0.229	0.028	<.001

Health information literacy	0.495	0.036	<.001

Adjusted R^2^	0.363	-	-

## Discussion

In support of the set hypothesis, a higher level of DSE was found among pwMS/E who reported more advanced HIL in terms of critical and risk-conscious literacy. In addition to a higher score for HIL, lower-level anxiousness or depression was associated with DSE in personal health management.

The findings are in line with the previous systematic review (Xu et al., 2018), focused on another chronic condition. The most consistent finding between the review and the current empirical study is that critical health literacy was emphasized as a key skill for the successful management of one's illness. This combined result suggests that, even among a more generalized group of chronic patients, those with HIL tend to be more confident in their ability to manage their illness also by utilizing digital services.

In Finland, a national Neuro register, including both the MS and epilepsy registries, is integrated into the electronic patient record systems of hospitals (Hämäläinen et al., 2024). The Finnish Neuro register has a patient interface through which patient-generated data can be shared with clinicians. In other words, pwMS/E in Finland may be relatively accustomed to managing their condition through digital service paths and interfaces. Indeed, eHealth systems should aim for positive user experiences because of their impact in supporting both HIL and DSE.

Furthermore, the principal component analysis revealed that DSE was the most distinct component in the data, which highlights the prominence of DSE in personal health management within the dataset. The significant role of self-efficacy in managing digitalization has been stressed in prior studies (Spreadbury et al., 2022). Self-efficacy has been found to be the strongest explanatory factor in a range of studies from eHealth usage to health care robotization (Britt & Hatten, 2016; Turja et al., 2020).

Although HIL is, by definition, more than an individual set of skills, the study design posed the risk of explaining competence by competence. To minimize overlapping with the concept of DSE in personal health management, HIL did not include a statement about basic, functional information-finding skills. Instead, HIL was operationalized as a critical and risk-related understanding of medical information. As a result, the study found that HIL, as the ability to evaluate and understand digital information about one's condition, is associated with DSE as the perceived, metacognitive confidence in one's competence in using eHealth services.

## Contextual Considerations

The findings of this study should be interpreted in light of the Finnish health care context. In Finland, the national Neuro register includes a patient interface that allows patients to share self-reported data with clinicians. As such, pwMS/E in Finland may be more accustomed to managing their condition through digital platforms than similar populations in other countries. This context presents a potential limitation to the generalizability of the findings, as prior exposure to structured digital health services may have positively influenced the levels of DSE and HIL reported in this sample.

Nevertheless, the identified association between HIL and DSE in a Finnish sample of neurological outpatients contributes to the growing body of evidence on this relationship. It supports the broader claim that chronically ill individuals with adequate HIL tend to feel more confident in managing their condition, also through digital means.
